# Restructuring Interlinked With Employer and Corporate Branding Amidst COVID-19: Embodying Crowdsourcing

**DOI:** 10.3389/fpsyg.2022.835017

**Published:** 2022-04-28

**Authors:** Raja Irfan Sabir, Mohammad Nazri, Muhammad Bilal Majid, Hamid Mahmood, Khurram Abbas, Sobia Bano

**Affiliations:** ^1^Faculty of Management Studies, University of Central Punjab, Lahore, Pakistan; ^2^Department of Business and Accountancy, University of Malaya, Kuala Lumpur, Malaysia; ^3^Faculty of Business and Management, Sultan Zainal Abidin University, Kuala Terengganu, Malaysia; ^4^Department of Business and Management, UniSZA, Kuala Terengganu, Malaysia; ^5^Commission on Science and Technology for Sustainable Development in the South (COMSATS) University Islamabad, Sahiwal Campus, Sahiwal, Pakistan; ^6^Gujranwala Institute of Future Technology (GIFT) Business School, GIFT University, Gujranwala, Pakistan

**Keywords:** restructuring, COVID-19 pandemic, crowdsourcing, employer branding, corporate branding

## Abstract

The COVID-19 pandemic is an unprecedented time in history. Surrounding this pandemic are many enormous uncertainties across the globe. Severe consequences have assessed for the incomes of almost 84% of employers and 68% of self-employed who are working and living in countries that are or have went through a phase of closing workplaces. Similarly, the global rate of unemployment is also expected to be increased in the coming years as 54% of employers worldwide are running their businesses in the hardest-hit sectors. All of these clearly show the uneven impact of the Coronavirus crisis (COVID-19) which will remarkably compound already present inequalities, difficulties, and vulnerabilities. The economic ramifications for 186 countries under the crunch of the COVID-19 pandemic is also considered tremendous for Pakistan. The core aim of this research was to test a new conceptual framework depicting the ramifications of restructuring processes carried out by management for their organizations amidst the COVID-19 pandemic on an Institute’s reputation as an employer brand. It also investigates the impact of perceived restructuring on a corporate brand promise made by the university or institute from the viewpoints of other key stakeholders and potential job seekers. The current study had proposed four hypotheses and according to the results of Structural Equation Modeling, the direct hypothesis based upon the relationship between restructuring and employer branding has been rejected. This study shows that restructuring and employer branding has a negative and insignificant effect on each other. The second direct hypothesis of the study that measures the effect of restructuring on corporate branding has been accepted. A corporate brand is a kind of a hub and it considers how an institute treats and deals with all of its stakeholders. It is different from employer brand as the perceptions of employees were the main focus. So, according to the results restructuring did not cause massive damage to the overall outlook of the institute. Furthermore, for the purpose of mediation analysis, the maximum likelihood method by bootstrapping was adopted to test the indirect hypotheses of the study. Crowdsourcing was introduced as a mediator in this study with restructuring, employer, and corporate brand all together in one framework, which is the novel aspect of this study. There are two indirect hypotheses and according to the results both of them did not show any insignificant results. Firstly, the study analyzed mediation among crowdsourcing, restructuring, and employer branding which was fully accepted as the results showed full mediation between these constructs. Secondly, the study analyzed mediation among crowdsourcing, restructuring, and corporate branding which was partially accepted as results showed partial mediation between these constructs.

## Introduction

The COVID-19 pandemic is an unprecedented time in history. Little is known about when this crisis will actually end, as until now, we have well known that it is not a temporary crisis. According to Ramkissoon (2020), the fundamental aspect of COVID-19 is to restrict travel across communities and the strong suggestion of staying at home to control its spread. Surrounding this COVID-19 pandemic are many great uncertainties across the globe (Venkatesh, 2020). Nobody could have ever imagined the impact of this pandemic a year or two ago and how many ways our lives can be affected by this crisis. In one way or another life has completely changed for everyone (Doyle and Conboy, 2020) from the beginning phase of the COVID-19 pandemic that was social distancing to the phase of strict lockdown in all areas. A series of unprecedented complications, impediments, and challenges have been thrown by the past few months to those who are protecting their health, those who are self-isolating, those on furloughs, those who are struggling between home-working and/or home-schooling, and those who recently faced unemployment (Hitchings and Maclean, 2020).

Under the considerations of the 17 Sustainable Development Goals Agenda, the motive is to collude to resolve advanced encounters to alleviate the social, environmental, and economic sustainability that paved the way toward priorities identification (United Nations Summit on Sustainable Development [UNSSD], 2015; Ramkissoon et al., 2020). A severe consequence is assessed on the incomes of almost 84% of employers and 68% of self-employed who are working and living in those countries that are undergoing the closure of workplaces phase. As per the nowcasting model of the International Labor Organization (ILO), a dramatic decline in global working hours has been found between the first and second quarter of 2020 ranging from 4.5 to 10.5% (Abulibdeh, 2020).

Similarly, the global rate of unemployment is also expected to be increased in the coming years as 54% of employers worldwide are running their businesses in the hardest-hit sectors. All of these clearly show the uneven impact of the Coronavirus crisis (COVID-19) which will remarkably compound the already present inequalities, difficulties, and vulnerabilities. The economic ramifications for 186 countries under the crunch of the COVID-19 pandemic is also considered tremendous for Pakistan. According to Ramkissoon (2021), numerous nations had closed their borders for months to restrain the virus from spreading. This pandemic has severely disrupted the normal economic activities between countries whether they are domestic economies or external relations. Some authors have suggested that the rate of impact of a globally negative economy will go beyond the Great Depression of the 1930s. During the Great Depression of 1930, there was a decline of 26.7% of Global GDP, and the rate of Global Unemployment was increased to 25% (Asian, 2020).

The outbreak has not proven dangerous only to the health of humans, but the survival and operations of every industry are at stake whether in a developed country or a developing one. According to Rydell and Kucera (2021), the COVID-19 outbreak restricts choices, restructuring behaviors, and procedures regarding services, even while lowering their alleged risk of experiencing such contagious disease. For instance, even developed economies find it hard to withstand the harsh effects of the COVID-19 pandemic (Singh et al., 2020). The COVID-19 pandemic hit developed countries first and then moved toward the developing countries that were not as advanced socially and economically. United Nations (UN), World Health Organization (WHO), and United Nations Development Program (UNDP) experts are concerned about the virus spread and its long-term impacts on underdeveloped nations (Hawker, 2020). These nations tend to strive for the betterment of their economy as they are poorer and are majorly dependent on their primary sectors (natural resources, agriculture, forestry, etc.). At times when the rich partners of their trading operations are also under the crunch of global pandemic (COVID-19) which causes them to shut their borders, then brutal pinch is being felt by developing nations and they will suffer great pain and losses.

According to the estimates of ILO, approximately 6.7% of global working hours will be wiped out during the pandemic phase (ILO-OECD, 2020). Apart from this, the outbreak has forced many organizations to alter the ways they work before forcing them to restructure, temporarily close operations; change their working patterns, and struggle to retain their operations. All of this is because of the high demand for health and occupational safety measures, Government decisions, and swap in consumer behavior as a result of which employees are facing unemployment, furloughs, weak social protection systems, and many more (OECD, 2020).

To support the operations of businesses and to support those in crisis Governments and management all around the world are taking precautionary measures and swift actions to keep the downturn shallow and preserve as many jobs as they can to not lose the trust or loyalty of employees (International Labor Organization [ILO], 2017). For this purpose, to design necessary measures the IOE (International Organization of Employers) and ITUC (International Trade Union Confederation) stand with Governments at national and multi-levels in solidarity (International Organization of Employers [IOE], and International Trade Union Confederation [ITUC], 2020).

The most common practices of this restructuring are furloughs, layoffs, work from home, reduced salaries, reduced or increased working hours, training about new methods and techniques, counseling, and redeployment (ILO-OECD, 2020). Properly planned restructuring according to the spur of the moment could put the company in line for recovery and cultivate a strong employer brand. Similarly, a poorly designed restructuring effect can reflect brutally on the overall image of the organization because a corporate brand promise was broken and as a result, the morale of workers will decline, there will be a lack of trust from the community, and a lack of skilled employees and experienced workers that might posses’ important knowledge of the workplace. All of these depend on how management and team leaders will carry out the process of restructuring (Vundla, 2012).

Present research aims to cover the gaps and make valuable contributions: (1), this paper seeks to establish by gauging the constructs on identical units of analysis. Researchers examined the effect of restructuring on the employer and corporate branding. (2), in the present research, crowdsourcing is presented as a mediator between restructuring and employer and corporate branding. This delivers an elucidation by way of in what way restructuring and employer and corporate branding are interconnected. The present model ought to lead to supplementary conjecturing by accenting instantaneously mediation that offers a cavernous lens to inspect the association entrenched in the research model.

## Hypotheses Development

### Restructuring and Employer Branding

Due to the COVID-19 pandemic that will remain looming in our rearview mirror for quite some time, there will be manifold outcomes everywhere including healthcare, economies, supply chain or labor markets, jobs, and home life as well. Consequently, there have been extensive changes to the nature of jobs. To reduce the adverse impacts of restructuring management can make adaptations like redeployments of employees, retraining, early retirements, etc. Some identified options for restructuring that are prevalent also include WFH, changed working hours, job sharing, furloughs, and wage subsidies. All of this is done by upper management for the survival of the operations of their firm, but if restructuring causes layoffs or dismissal of employees then it can harm the reputation of an organization along with negative consequences for the community (International Labor Organization [ILO], 2017). One gigantic change that employees have to face is of course, without any doubt, job loss. The process of restructuring is mostly associated with negative impacts on the wellbeing of employees, and the best explanation for this negative consequence might be downsizing (Jong et al., 2016). The adverse changes also include those knowledge workers which are now working from home and are expected to do so for longer periods. It is worth noting that, throughout history, a large percentage of workers have never yet worked virtually for an extended time (Venkatesh, 2020).

The magnitude of people who lost their jobs in these crucial times has stretched to staggering levels. The consequence of which is diverse economic concerns and hopelessness on the part of workers along with adverse reactions (Kochhar, 2020). Besides, those who did not lose their jobs and fortunately remain employed have to face substantial changes as per new technology (Morris, 2007). To the best of our knowledge, the relationship between restructuring causing layoffs does not have much empirical study yet. But, the available literature shows potential effects between brand value and restructuring (Campos-García and Zúñiga-Vicente, 2018).

Concerning restructuring, employer branding yields vital awareness and information about an organization’s benefits to potential and current employees about working with such an employer (Kashive et al., 2020). By knowing it is dealing in the context of restructuring, employer branding will be affected remarkably. In some qualitative studies, it was also assessed that higher retention rates of workers can be achieved with stronger employer branding (Tanwar and Prasad, 2016). A number of audiences are watching and observing organizations’ treatment of those who are working for them. They are considered to be vital stakeholders including active or passive job seekers, future potential contenders, and the general public as well (Smart Dreamers Team, 2019). Moreover, when a job seeker needs to decide to choose an organization to work with, the image or value created by a strong employer brand will become most significant regarding the job and treatment of the employer of current employees as it provides differential ascendency as compared to weaker employer brands. Jobseekers are most afraid of uncertainties that might lead them to end up unemployed or into any other severe situations. Hence, strong employer brands automatically discharge these uncertain risks for job seekers to decide.

Furthermore, the magnitude of attracting skilled job seekers is the expression of a good employer brand (Kissel and Büttgen, 2017). However, there are significant benefits that come with employer branding, but still, it can be damaged and adversely affected by turbulent events under restructuring such as layoffs. As a result, there will be disruptive changes in the perceptions of those who are working inside the organization, potential employees, former workers, and external stakeholders as well. These sudden uncertainties experienced by them can reduce their loyalty, trust, commitment, enthusiasm, and satisfaction. In the long run, it causes present employees to seek another workplace and for potential employees to decide between pursuing jobs elsewhere. In all aspects, it deteriorates employer brand value for those who will arrive in the future to work, those who remain, those who had to leave, and those who are somehow related to the organization (Garas et al., 2018).

Vulnerabilities deriving from restructuring across the world are also visible in post-secondary systems as well. The uncertainty of a future education system that is more flexible and resilient is the demand of society in this era. Where the virtual environment is firmly established as saving students and teachers from sitting idly during the pandemic there are many educators concerned about the troubles which followed this crisis. Many are facing troubles in swapping to online teaching or becoming unemployed. It is also accompanied by fewer tuition fees or more job losses decreasing academics’ reputation (Houlden and Veletsianos, 2020). Lastly, these job changes also affect employees in many dramatic ways and their job outcomes as well. Hence, management needs to work on this like Google and Amazon are, thinking strategically to decrease the stress levels of their employees related to restructuring or any sudden uncertain change. These companies are giving their employees a day off to “unplug” and recharge (Bariso, 2020). So, we have found out that the employer brand during restructuring is considered to be most critical and at the same time vulnerable (Fernandes, 2019).

**H1:** Restructuring significantly and positively affects employer branding.

### Restructuring and Corporate Branding

A corporate brand does not only mean to have logos, names, or effective taglines, etc., it derives from multiple factors such as how an organization deals or interacts with the public or its employees. Cooperation of internal and as well as external stakeholders is needed for the accomplishment of goals and to align them with corporate promises and values (Agbo and Asadu, 2016). The firm’s overall image won’t remain the same all of the time; it will continuously evolve based on those choices like in this study’s context of restructuring that management makes to create value for its stakeholders. But it is critical for choices to be perceived as appropriate to boost the image of a company. These might include choices of introducing innovative techniques to employees or other market-related actions. According to Watson and Popescu (2021), COVID-19 restructured attitudes, activities, standards, potentials, individualities, feelings, conviction, and assignation, and paved the way to transform traditions in terms of emotional risk perception. But some choices can be perceived as controversial by stakeholders and will drown the firm’s reputation. These might include the choices that managers make for their self-interest or to favor specific stakeholders’ interests over others, as a result, they may face some reputational penalties (Bednar et al., 2015).

Usually, managers consider downsizing a feasible practice to improve a company’s performance, reduce costs and refine its competitive position, but it risks and of course harms the corporate image of the firm. Also, while downsizing might cause short-term positive impacts they are usually short-lived (Carriger, 2018). The most important resource a firm has are its human resources that share important knowledge, capabilities, and skills. A firm can never imitate its human resource because of its ability for efficient output creation. Indeed, employees would think about the risks of job security which also cause a lack of commitment on their part followed by stress; they always stand against this type of activity, considering them cruel (Rophae, 2020).

Moreover, the capability of creation and innovation can also be damaged through restructuring because it will disrupt informal communication and create an atmosphere of unfairness and a culture of distrust ultimately deteriorating the information sharing process at the workplace. In sum, according to previous research, there are many negative implications of downsizing on customers, employees, investors, and suppliers. Consequently, because of downsizing the future prospects of the company will become uncertain in the eyes of stakeholders and they consider the firm less favorable due to restructuring (Schulz and Johann, 2018). In critical cases like layoffs, careful consideration about brand, employees, and customers before that layoff restructuring is required. It may also require a communication strategy, and a competent one is important to handle the whole process with precision before and after as well (Labovich, 2016). Reorganization might be considered favorable by owners and stockholders of the company but for current and potential employees, it creates a negative corporate image. It also decreases the overall corporate image of the firm in the eyes of customers if by any chance the quality of products and services is not up to the mark due to the reorganization (Acevedo, 2020). A situation of puzzlement and disorder will take place within and outside of the organization if there is continuous restructuring (Akumu and Nzulwa, 2018).

**H2:** Restructuring significantly and positively affects corporate branding.

### Crowdsourcing, Restructuring, and Employer Branding

In this tumultuous period, the Coronavirus crisis (COVID-19 pandemic) not only forced firms to alter the ways they perform their tasks but also changed what type of work is performed and how to perform that work (Jesuthasan et al., 2020). The worldwide economy was disturbed through this pandemic and health care affected, triggering uncertainty while inciting fear amongst billions of people (Valaskova et al., 2021). In this regard asking employees about ideas or making them take part in decision-making can be prove helpful. Outsourcing some pieces of work from a large group of people outside the workplace has become acceptable in the world of modern business and is also considered a salient decision support strategy. A case was assessed through media reports of IBM that they planned a vast restructuring process which included lots of layoffs. They planned to conduct a fire and re-hire strategy, but on the end of the employees that did not seem accurate.

To avoid such circumstances, the best option is to go for employee-based crowdsourcing where employees can take part in decision-making and then in further job-related tasks after restructuring. Managers need to understand that each employee working in their firm is also a consumer themself and holds valuable knowledge. By accepting this, they can crowdsource their employees’ knowledge and experience to another level within the firm (Ivanov, 2012). Also, in this black swan era (COVID-19 pandemic), understanding which type of strategy will best support the organization and employees on the part of management is vital, especially to support diverse technology-related work decisions (Sykes and Venkatesh, 2017) because they will help companies in interventions to support their key internal stakeholders during tumultuous times for now and for the future as well (Sykes, 2015).

To open the floor by employers for employees to gather ideas that how a company would restructure during the COVID-19 pandemic with the help of the Crowdsourcing phenomena is overwhelming. But it does not simply mean that employers are not efficient and are losing their control. Crowdsourcing does not have to be parallel to chaos but it signals that employees are valuable to the company and employers care about their workers and consider them as assets of the firm. Moreover, that the company does not fake their care by only saying this to employees; it is a way of showing that they consider them worthy (Tarki et al., 2020). To evaluate important employee value propositions (EVPs), an employer also uses crowdsourced sites like Glassdoor for future decision-making (Dabirian et al., 2016). During a pandemic where every firm has to restructure, they can evaluate value propositions to make the best decisions about how to restructure.

Consequently, employer branding consists of two main important stakeholders, which are internal employees and external potential candidates. Internal employees are the ones having accurate information about the workplace and the treatment by the employer within them. But external job candidates can only rely on social media word of mouth from existing workers for information. In such cases, to make it better for employers about what employees feel, encounter, and observe they can understand internal employees’ needs more closely (Suen et al., 2020) and then readjust their restructuring process accordingly. Also, with the development of social sites, many people rely on the opinion of peers and their feedback even on social media platforms like Twitter, Instagram, Facebook, LinkedIn, and YouTube. Workers share their experiences through word of mouth on these platforms and open new ways for employer branding for companies (Kashive et al., 2020). As a result, this will shape the perceptions of potential employees about employer branding during the COVID-19 pandemic.

Some authors also estimate that even when this pandemic is over, the changes that occurred during the pandemic might become permanent or needed in post-pandemic periods in workplaces and jobs because companies are striving to become more flexible (permanent WFH) to face any other uncertain situation in the future (Byers, 2020; Goldstein, 2020; Kelly, 2020). Up until now it is clearly evident that in these times of change (restructuring) in organizations everybody is in a state of bewilderment at how to tackle this restructuring event that is the requirement for firms to be able to survive. Managers are not really sure of what decision to go for and what will be the consequences of those decisions on their organizations’ reputation as an employer or on their employees. During these tumultuous times, the introduction of a new strategy to be adopted is the requirement. This crowdsourcing is introduced in this study to be used by leaders as a means to get information regarding restructuring and in the end, give cues of good treatment as well ultimately putting employer reputation in the line of recovery that was damaged by continuous or sudden restructuring events. Whether crowdsourcing can help in restructuring and recovering employer branding will be identified in this study. The goal is to see that if managers put crowdsourcing before the restructuring process, then it will give fruitful results.

**H3:** Crowdsourcing mediates the significant and positive relationship between restructuring and employer branding.

### Restructuring, Crowdsourcing, and Corporate Branding

Among the variety of factors that contribute to the organization’s reputation, social media is also the vital one. As the literature shows that social media also have the major power in its hands by which the image of the firm can be shaped. It also illuminates that in today’s world, reputation is the most important phenomenon and because of the internet-prone generation it tends to change quickly. Hence, the strong strategic managerial decision-making process manifests the corporate brand image (Mcsweeney, 2019). And in this study, crowdsourcing to be implemented in the process of decision making is also the goal. Management are required to carefully handle restructuring because it can affect organizations’ corporate image and the implementation of any change in their organization in times when it is necessary. Promise management is the main concern of a corporate brand which consists of internal and employer branding as its two main pillars. Where on one hand, employer brand is all about differentiating an employer in terms of value creation for employees from other employers in order to retain them and to attract those who will easily align themselves with the corporate values as well. On the other hand, incorporating internal branding management is making sure to communicate their corporate values in the best possible way to employees for them to represent and boost their engagement as well (Foster et al., 2010). Internally dealing with stakeholders is also very important in this case, such as for employees to get to know why any specific change is required or why management decides to go for layoffs, etc. as this practice can also create strong relations of stakeholders with the firm.

Also, corporate branding reduces the risks of many uncertainties for customers and employees. The available information regarding the firm’s activities and operations from the basic perceptions of external observers in terms are corporate image. External stakeholders use news regarding the firm’s operations, dealings, press releases, achievements, financial performance, and quarterly results along with its advertising as cues to evaluate the upcoming prospects and the status of the organization. But these signals are delivered to stakeholders using some information intermediaries. These channels such as magazines, research reports, social media sites, chat rooms, and news create an overall judgment about the corporate brand (Schulz and Johann, 2018). So, the information regarding continuous or sudden restructuring can also spread very easily. But the case that is focused on in this study is whether the information that is spreading about the institute regarding restructuring can be good. For instance, good information can go around everybody if managers use efficient strategy because in this way outlook of the firm will never be easily harmed.

Any firm undergoing restructuring must work hard to keep its image intact because, in today’s world where the online community is the most important one, it is only a matter of seconds and a single angry tweet to put any firm’s image under fire (Roberts, 2020). It is acceptable that during this pandemic tenure some firms have little option but to go for layoffs and furloughs which are considered as horrible actions to be taken. But a very important thing was highlighted: that how any organization makes their employees leave is very crucial for the firm’s image itself, for its remaining employees, for potential employees, for consumers, and other stakeholders as it will influence the institute’s image. It is also very important that how managers make these restructuring decisions will influence the stories that will be told about the company altogether with the triumph in this blighted era and beyond.

It is no doubt that layoffs are a kind of restructuring which comes as a last resort for management, and if they share it with stakeholders precisely using crowdsourcing and giving cues about why management needed to make such a decision, they might support the firm. But in cases where it is the result of management’s self-interest then it will drastically affect the company’s corporate reputation because in today’s world where crowdsourcing and social communities are substantially prevailing people are observing companies’ every activity very closely. Additionally, by showing that they care about the concerns of employees, companies ensure restructuring is fair, and by involving people to give their opinions firms can maintain corporate brand identity (Frauenheim, 2020).

Furthermore, the available information about the company on social networks not only affects employer brand attractiveness but will also influence corporate brand image as well. Long before the beginning of the hunt for a job at a great workplace individuals might have already formed some basic image and developed a general perception regarding the organization (Kissel and Büttgen, 2017) from the information prevailing on the web or by word of mouth that comes into the context of crowdsourcing. It is also noteworthy by management in this uncertain pandemic that corporate brand is also about quality, history, social responsibility, reputation as an employer, success on financial terms, and behavior with stakeholders. Using communication channels, they can disseminate these things as well (Agbo and Asadu, 2016). So, crowdsourcing as a mediator is used in this study on the relationships between restructuring and corporate branding to identify its effect on both. A corporate brand is not merely about having a good name, a variety of products and services, or attractive logos, but it is also about how an entity treats its stakeholders. And during times of restructuring, organizations’ images are very unstable or unpredictable both inside and outside of the firm. In such situations implementing a strategy like crowdsourcing and using the information, it gives in the process of restructuring might help an institute to save its corporate image.

**FIGURE 1:**
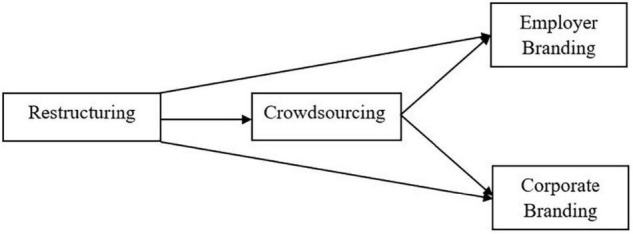
The research framework.

**H4:** Crowdsourcing mediates the significant and positive relationship between restructuring and corporate branding (Figure 1).

## Research Methodology

### Nature of Research

The nature of our study is descriptive and explanatory in the light of its objectives which are to discover, manifest, and measure the causal relations of the defined variables as it is best in explaining the relationships of many important factors. Whereas, exploratory nature is a kind of initial research where a researcher has to begin with an idea and seeks to learn more. This nature covers almost new ideas, angles, or topics (Given, 2008).

### Research Strategy

Furthermore, about the research strategy, the survey method would be used because it is best suited for this study under the quantitative method (Cahill, 2018). A Self-Administered Questionnaire Survey was conducted to collect data in order to and to evaluate the cause-and-effect relationships of the constructs for this study in the education industry using a cross-sectional time horizon because the investigation in this study revolves around a certain time frame. For data analysis SEM technique (Fan et al., 2016) through AMOS software has been used.

### Sampling Technique

There are so many useful techniques available to be applied in research in order to collect data from those who in our perception and knowledge are the best relevant audience for our study. As we are targeting the core employees of the education industry of Lahore in this study and our calculated sample size is 250 (Hair et al., 2006) so we collected data from teachers of the education institutes. We could not access each respondent by ourselves in the given time frame and as well as it is not humanly possible to reach everybody living in such a big city whenever you need it, so we have to adopt a sampling technique. The sampling method that was used in this study is non-probability convenience sampling in which we selected respondents easily available at the time frame of this study to participate (Lavrakas, 2008). Convenience sampling was used based on some criteria met by the target audience like accessibility at a given time frame, geographical proximity, or willingness by the participant to participate as well (Etikan, 2016). Furthermore, there are many cases in which this sampling technique is the only option to go for. As in this study, it was the best option amidst the surrounding danger and risk of the COVID-19 pandemic because the study is considering those whom we were able to access easily using our contacts (Saunders et al., 2015). The best example of this is given by James, who collected data using convenience sampling from a number of students (Herring, 2010).

### Data Collection Method

Most countries were facing lockdown during the first half of the year 2021 and people were in a panic situation due to layoffs, cuts in salary, as well as physical suffering from COVID-19. Hence, people were not responding to surveys or questions regarding their job or institutes, so researchers had to focus only on Pakistan where they had strong networking. Moreover, the data were collected from private educational institutes in Lahore during the months of April 2021 to June 2021. The reason to be limited to the one city of Lahore besides its Provincial Capital status and metropolitan nature is that a large proportion of universities are situated in Lahore. Moreover, the city has now exceeded nearby Karachi in terms of population, and the estimated population of Lahore in 2021 is 13,095,166 consisting a diverse population with diverse backgrounds, languages, and religions (World Population, 2021). Furthermore, Lahore city is considered the Educational Capital of Pakistan for having a larger number of colleges and universities than any other city. Population-wise, Punjab is the biggest province (Division, 2018) among other provinces of Pakistan having the highest population in Lahore city. According to the HEC total, there are 78 recognized Universities in Punjab of which 38 are located in Lahore city (HEC). The sample size was based on the formula given by Hair et al. (2006) in which the total number of items were multiplied by 10. Hence, the data was collected from 390 respondents because the total number of items is 39 using self-administered questionnaires. The questionnaire includes 39 questions (Table 1), and 3 of them will be demographic ones.

**TABLE 1:** Research instrument.

Variables	Name of measures	Number of items	References
Independent variable	Restructuring	7	Brand and Wilson, 2000
Dependent variable-1	Employer branding	14	Cahill, 2018
Dependent variable-2	Corporate branding	5	Koskela, 2010
Mediator	Crowdsourcing	10	Kim et al., 2012

The items were then processed in three important steps. The three steps are pre-testing, pilot testing, and then final data collection. In pre-testing, content and face validity will be checked from at least 20 participants of which 2 will be considered experts (Berg, 2014). After receiving suggestions from the pre-testing process the survey is good to go for further pilot testing in which 100 responses will be collected and then reliability analysis will be conducted on that data. After pilot testing, there were some items that were needed to be excluded and then the questionnaire consists of 25 items. Then in the last, after collecting the whole data for this study confirmatory factor analysis will also be conducted.

### Methods

A two-steps approach was applied in this research for the model’s reliability and validity assessment (Anderson and Gerbing, 1988). Initially, researchers conducted Confirmatory Factor Analysis (CFA). The goodness of fit indices represented the model to be in an acceptable range [χ^2^ (293.037) 5 186, χ^2^/df 50.325, goodness-of-fit index (GFI) 0.933, adjusted goodness-of-fit index (AGFI) 0.921 (MacCallum and Hong, 1997), normed fit index (NFI) 0.932, relative fit index (RFI) 0.944, comparative fit index (CFI) 0.911; tucker lewis index (TLI) 0.958; root mean square error of approximation (RMSEA) 0.038, parsimony comparative fix index (PCFI) 0.831, parsimony goodness of fit index (PGFI) 0.872, and parsimony normed fit index (PNFI) 0.799] by Hu and Bentler (1999). After that CR, Discriminant validity, and Convergent validity were assessed. According to Hair et al. (2011), the rule of thumb for CR is to be greater than 0.70. The discriminant validity was tested through constructs’ correlation and the square root of AVE whose threshold value is 0.85. The convergent validity was assured via factor loading (0.70) and AVE (0.50) (Hair et al., 2011). Findings revealed that the present research meets all requirements. Finally, for the purpose of checking common method variance, researchers employed the test of Herman single factor as suggested by Podsakoff et al. (2003).

## Data Analyses and Results

### Pre-testing

In pre-testing, content and face validity was checked from 20 participants of which 2 were considered experts. The questionnaire was given to twenty teachers from the target audience and some gave their valuable suggestions. At this stage of the study, some items were removed from the questionnaire. After this, 25 items remained for collecting the data from the target audience. After receiving suggestions from the pre-testing process and implementing the necessary changes the survey was deemed good to go for further testing.

### Demographics

In this research, the demographic section of the questionnaire was divided into gender, age, education, and income of the participants. Of the respondents to answer this study, 34.4% were men and 65.6% were women. There were 72.4% of respondents whose age was below 30 years, 16% of respondents whose age was between 31 and 40 years, 8.8% of respondents whose age was 41–50 years, and 2.8% of respondents whose age was 51 years or more. We found 23% of respondents were graduates, 47% of respondents had a master degree, and 30% of respondents had a MS/MPhil degree. Regarding income, 39% of respondents had an income between 30,000 and 40,000 PKR, 27% fell in-between 41,000 and 50,000 PKR, 19% of respondents had an income between 51,000 and 60,000 PKR, and 15% of respondents had an income of 61,000 or more.

### SEM Analysis

#### Standardized Estimations

**FIGURE 2:**
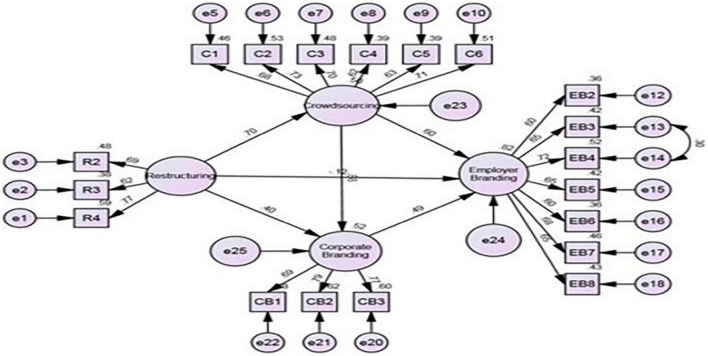
SEM (standard estimates).

For the current study, a standardized estimation model (Figure 2) was applied; a relationship between the variables—restructuring, employer branding, corporate branding, and crowdsourcing—was created through AMOS analysis. The below graph shows the visual output of path analysis by displaying the effects of exogenous constructs caused on endogenous constructs;

For this analysis, there are standard factor loadings and R square values for all the variables. The standard regression estimations of all independent variables are linked with all dependent variables. In this structural model, the value of R square is 0.82 for employer branding and 0.52 for corporate branding. The value of R square must be 0.40 or greater. It determines that 82% of change occurs in the endogenous variable (employer branding) by means of exogenous variable (Restructuring). Similarly, 52% change occurs in corporate branding due to exogenous variables (Restructuring) (Nawal et al., 2020). To check the model fitness, different indices were considered in SEM analysis.

In the above Tables 2, 3, regression weights were shown for standardized estimations. The path analysis shows the acceptance and rejection of the proposed hypotheses for this study. Beta values and significance levels were considered for this purpose. In the above table, highly significant *P*-values were shown by this symbol ^***^ which means 0.000, overall *P*-value less than 0.05 is acceptable. *P*-value plays important role in the acceptance or rejection of any hypothesis. The analysis explains that H1 which measures that restructuring significantly and positively affects employer branding is not accepted as H_1_: β = −0.12, *t* = −1.57, *p* = 0.224, which means there is a negative and insignificant relationship between the two. This finding is consistent with that of the previous studies (Garas et al., 2018; Fernandes, 2019; Houlden and Veletsianos, 2020). Standard estimation displays that H2 which measures that restructuring significantly and positively affects corporate branding was accepted as H_2_: β = 0.40, *t* = 4.16, *p* = 000, which means restructuring is positively and significantly affecting corporate branding of education institutes in Pakistan. This result is inconsistent with that of the previous studies (Akumu and Nzulwa, 2018; Schulz and Johann, 2018; Acevedo, 2020). The reason for such contradiction could be differences in culture (Małgorzata et al., 2021), brand loyalty (Muhammad, 2021), strong brand image (Anas et al., 2021), etc.

**TABLE 2:** Structural equation modeling (SEM) regression weights (standardized estimations).

Causal paths	Estimates	S.E	C.R	*P*	Hypotheses
CB <--- R	0.40	0.096	4.16	***	Accepted
EB <--- R	–0.12	0.076	–1.57	0.224	Rejected

This symbol *** means value is 0.000 and P value is highly significant.

**TABLE 3:** SEM regression weights.

Causal paths	Estimates	S.E	C.R	P	Hypotheses
C <--- R	0.70	0.080	8.75	***	Accepted
CB <--- C	0.88	0.108	5.46	***	Accepted
CB <--- R	0.40	0.096	4.16	***	Accepted
EB <--- C	0.60	0.101	5.45	***	Accepted
EB <--- R	–0.12	0.076	–1.57	0.224	Rejected

This symbol *** means value is 0.000 and P value is highly significant.

### Mediation Analysis

For mediation analysis, five paths were created which are shown in the table below. Their beta values and *p*-values are also mentioned in the table. In this study, there is one construct that plays the role of the mediator and that is crowdsourcing.

#### Mediation Analyses With DV1

To test the mediation relationships maximum likelihood estimation bootstrapping was applied using 1,000 bootstrap samples, 95 percent bootstrap interval, and 95 percent bias-corrected confidence (Rehman et al., 2021) (Table 4). In the above table, the mediation indirect regression line is displayed. β = 0.42 (0.70*0.60) with *p*-value = 0.000 was generated, which was significant. Moreover, the direct effect was insignificant as β = −0.12 with *p*-value = 0.224 which is not accepted as significant. According to Awang (2015) for the existence of mediation path A and path B needs to be significant. Both of these paths are significant which means that there exists mediation. Moving toward path C then if it is significant then there will be partial mediation and if it is insignificant then there will be full mediation. Hence, full mediation has been proved because paths A and B were significant and path C was insignificant. Crowdsourcing fully mediates the positive and significant relationship between restructuring and employer branding, which is consistent with previous research (Kashive et al., 2020; Suen et al., 2020).

**TABLE 4:** Mediation analyses with DV1.

Relationship path	Standardized beta	*P*-value	Results
Path A = R → C	0.70	0.000	Significant
Path B = C → EB	0.60	0.000	Significant
Path C = R → EB	–0.12	0.224	Insignificant
A*B (0.70*0.60) A*B > C	0.42	Full mediation because direct effect “Path C” is insignificant	

This symbol * means value is 0.000 and P value is just significant.

#### Mediation Analysis With DV2

For the second indirect hypothesis, three paths were created (Table 5). For instance, the first path was between an independent and mediating variable which is called “Path A.” The second path was created between mediating and the second dependent variable of this study which is called “Path B.” Lastly, a path that is known as “Path C” was created between the independent and second dependent variable of this study.

**TABLE 5:** Mediation analyses with DV2.

Relationship path	Standardized beta	*P*-value	Results
Path A = R → C	0.70	0.000	Significant
Path B = C → CB	0.88	0.000	Significant
Path C = R → CB	0.40	0.000	Significant
A*B (0.70*0.88) A*B > C	0.616	Partial mediation because direct effect “Path C” is significant	

This symbol * means value is 0.000 and P value is just significant.

To test the mediation relationships maximum likelihood estimator bootstrapping was applied using 1,000 bootstrap samples, 95 percent bootstrap interval, and 95 percent bias-corrected confidence (Rehman et al., 2021). In the above table, the mediation indirect regression line is displayed. β = 0.616 (0.70*0.88) with *p*-value = 0.000 was generated that was significant. Moreover, the direct effect was significant as β = 0.40 with *p*-value = 0.000 which is accepted as significant. According to the results, all three paths were significant. Hence, partial mediation has been proved (Awang, 2015). Crowdsourcing partially mediates the positive and significant relationship between restructuring and corporate branding, which further strengthens the findings of earlier studies (Kissel and Büttgen, 2017; Frauenheim, 2020).

### Discussion on Findings

There exists a relationship of crowdsourcing that mediates the relationship between restructuring, employer, and corporate branding in this study. Coronavirus and the COVID-19 pandemic is considered a black swan era because it changes everything and is surrounded by many uncertainties. The major purpose of this study was to scrutinize the ramifications of the restructuring process carried out by the top management for their organizations’ running operations amidst the COVID-19 pandemic, on firms’ reputation as employer brands from the perceptions of employees. This study contributes altogether to business management and practice as well. The findings provide many deep insights to the organization, management, and team leaders on how to tackle the restructuring of jobs among employees without risking the corporate and employer brand image. It also illuminates how Crowdsourcing can be used to generate ideas from stakeholders and how it will contribute to the formation of the firm’s image as a brand. This study is the first to examine the issue of restructuring during the COVID-19 pandemic in the context of the education sector. It is also the one to fill the room and reduce the gap between specific instances of restructuring upon the employer and corporate branding. Moreover, it also contributes to the emerging research on the influence of redesigning job events on firms’ reputations. It examines how a particular event of restructuring like downsizing, furloughs, and others might affect stakeholder perception. Furthermore, this study also has some important implications for managers as well.

### Implications of Study

The study results will help management to understand how they can improve their image in minds of employees and the general public by using the wisdom of the crowd through many means amid the COVID-19 pandemic or other pandemics. Team leaders can also use crowdsourcing on a regular basis to improve their image as an employer brand or to increase the trust and loyalty of their employees. Also, the study will create awareness among general public about the outlook of institutes at the time of choosing one to work with. Employers can view crowdsourced ratings and also generate word-of-mouth on the web to give a sign of employer branding (EB) to retain and attract talents. They can also identify important EVPs that satisfy their employees and also those that they will not consider satisfactory. Managers can also get to know about any issue/misunderstanding that was prevailing and then communicate to solve it. They can understand that employees are uncomfortable due to sudden restructuring and can inform them why managers had to take actions like downsizing events or they can ask employees to give their ideas and feedback for them to work with ease. Also, the study will become the foundation for institutes to formulate restructuring policies and strategies to be used effectively by managers.

### Limitations and Future Directions

The study results cannot be generalized to other sectors or regions as the study and its application might not provide the expected results. The study will only be applicable in pandemics and not in normal conditions. Time tenure for the study was only limited to the time given by the university to complete the research leaving it to be cross-sectional, but if a longitudinal study from the beginning to the end of the pandemic is carried out then it will give accurate results. The study is only limited to one industry which is education. The respondents for this study are limited to one group of participants from the education industry, that being teachers. The study is conducted in Pakistan, so the results will be best applicable in Pakistan only and the framework might not give desired or similar results in another country. Also, the data was collected from one city only, that being Lahore. A bigger sample can be adopted in future studies. The study can be carried out in a longitudinal-time horizon for better insights. This study only focused on teachers as respondents, but in the future other authors can go for other diverse groups of the target audience as well. In the education sector, there are non-teaching staff as well who also faced restructuring. So, future authors can look upon them as well. Other variables in a similar domain can also be incorporated into the proposed framework such as internal branding, employee engagement, ethical branding, and others. The cross-cultural study can also be applied by future researchers. Mixed or multi-methods can be used in the future as well.

## Data Availability Statement

The raw data supporting the conclusions of this article will be made available by the authors, without undue reservation.

## Ethics Statement

Ethical review and approval was not required for the study on human participants in accordance with the local legislation and institutional requirements. Written informed consent was not required from the participants to participate in this study in accordance with the national legislation and the institutional requirements.

## Author Contributions

RS found the research gap and the problem statement. MN found the research model and the title of the manuscript. MM conducted the literature review and analysis. KA wrote the background of the study. SB wrote the conclusion and implications section and proofread the draft. All authors read and approved the final manuscript.

## Conflict of Interest

The authors declare that the research was conducted in the absence of any commercial or financial relationships that could be construed as a potential conflict of interest.

## Publisher’s Note

All claims expressed in this article are solely those of the authors and do not necessarily represent those of their affiliated organizations, or those of the publisher, the editors and the reviewers. Any product that may be evaluated in this article, or claim that may be made by its manufacturer, is not guaranteed or endorsed by the publisher.
